# Feasibility of laparoscopic versus open pancreatoduodenectomy following neoadjuvant chemotherapy for borderline resectable pancreatic cancer: a retrospective cohort study

**DOI:** 10.1186/s12957-023-03277-2

**Published:** 2024-01-02

**Authors:** Zheng Li, Qifeng Zhuo, Borui Li, Mengqi Liu, Chen Chen, Yihua Shi, Wenyan Xu, Wensheng Liu, Shunrong Ji, Xianjun Yu, Xiaowu Xu

**Affiliations:** 1 Department of Pancreatic Surgery, Fudan University Shanghai Cancer Center, Shanghai, 200032 China; 2 Department of Oncology, Shanghai Medical College, Fudan University, Shanghai, 200032 China; 3 Shanghai Pancreatic Cancer Institute, Shanghai, 200032 China; 4 Pancreatic Cancer Institute, Fudan University, Shanghai, 200032 China

**Keywords:** Pancreatic ductal adenocarcinoma, Neoadjuvant chemotherapy, Laparoscopic pancreatoduodenectomy, Feasibility, Prognosis

## Abstract

**Background:**

There is no evidence supporting the feasibility of laparoscopic pancreaticoduodenectomy (LPD) compared to open pancreatoduodenectomy (OPD) following neoadjuvant chemotherapy (NACT) for pancreatic ductal adenocarcinoma (PDAC).

**Methods:**

The clinical data of consecutive patients with borderline resectable PDAC who received NACT and underwent either LPD or OPD between January 2020 and December 2022 at Fudan University Shanghai Cancer Center was prospectively collected and retrospectively analyzed.

**Results:**

The analysis included 57 patients in the OPD group and 20 in the LPD group. Following NACT, the LPD group exhibited a higher median CA19-9 decrease rate compared to the OPD group (85.3% vs. 66.9%, *P* = 0.042). Furthermore, 3 anatomically borderline PDACs in the LPD group and 5 in the OPD group were downstaged into resectable status (30.0% vs. 12.3%, *P* = 0.069). According to RECIST criteria, 51 (66.2%) patients in the entire cohort were evaluated as having stable disease. The median operation time for the LPD group was longer than the OPD group (419 vs. 325 min, *P* < 0.001), while the venous resection rate was 35.0% vs. 43.9%, respectively (*P* = 0.489). There was no difference in the number of retrieved lymph nodes, with a median number of 18.5 in the LPD group and 22 in the OPD group, and the R1 margin rate (15.0% vs. 12.3%) was also comparable. The incidence of Clavien-Dindo complications (35.0% vs. 66.7%, *P* = 0.018) was lower in the LPD group compared to the OPD group. Multivariable regression analysis revealed that a tumor diameter > 3 cm before NACT (HR 2.185) and poor tumor differentiation (HR 1.805) were independent risk factors for recurrence-free survival, and a decrease rate of CA19-9 > 70% (OR 0.309) was a protective factor for early tumor recurrence and overall survival.

**Conclusions:**

LPD for PDAC following NACT is feasible and oncologically equivalent to OPD. Effective control of CA19-9 levels is beneficial in reducing early tumor recurrence and improving overall survival.

**Supplementary Information:**

The online version contains supplementary material available at 10.1186/s12957-023-03277-2.

## Introduction

Pancreatic ductal adenocarcinoma (PDAC) is one of the most aggressive tumors with a rising incidence and ranks as the fourth leading cause of cancer-related deaths [1]. Radical resection remains a potential curative treatment option for selected patients. According to the Miami international evidence-based guidelines, minimally invasive resection has been shown to be feasible, safe, and oncologically equivalent for PDAC patients compared with open surgery [2–7]. Furthermore, studies have demonstrated that minimally invasive pancreatomy is associated with improved overall and disease-free survival outcomes [8].

However, it is important to note that only 15 to 20% of PDACs are eligible for upfront surgery at the time of initial diagnosis [9]. For borderline resectable PDACs, studies have confirmed the oncological benefits of neoadjuvant therapy than upfront surgery, such as improved rates of margin-negative resection and a decreased incidence of lymph node metastases [10–13]. Additionally, short-course neoadjuvant therapy has been shown to improve postoperative survival [14–16]. These findings strongly support the use of short-course neoadjuvant therapy in borderline resectable PDACs, aligning with the recommendations provided by the National Comprehensive Cancer Network guidelines.

Neoadjuvant therapy can lead to severe fibrosis in the localized tumor tissue, which may hinder dissection and increase the risk of dangerous and bloody surgery. Furthermore, most borderline resectable PDACs have a large diameter and are in close proximity to major blood vessels, making the surgical procedure more complex and challenging. To date, there is insufficient evidence to determine the feasibility and safety of minimally invasive pancreatectomy compared to open surgery after neoadjuvant therapy [5].

This study aims to assess the feasibility of laparoscopic pancreatoduodenectomy (LPD) compared to open pancreatoduodenectomy (OPD) for borderline resectable PDACs following neoadjuvant chemotherapy (NACT) in a prospectively maintained database.

## Methods

### Study population

**Fig. 1:**
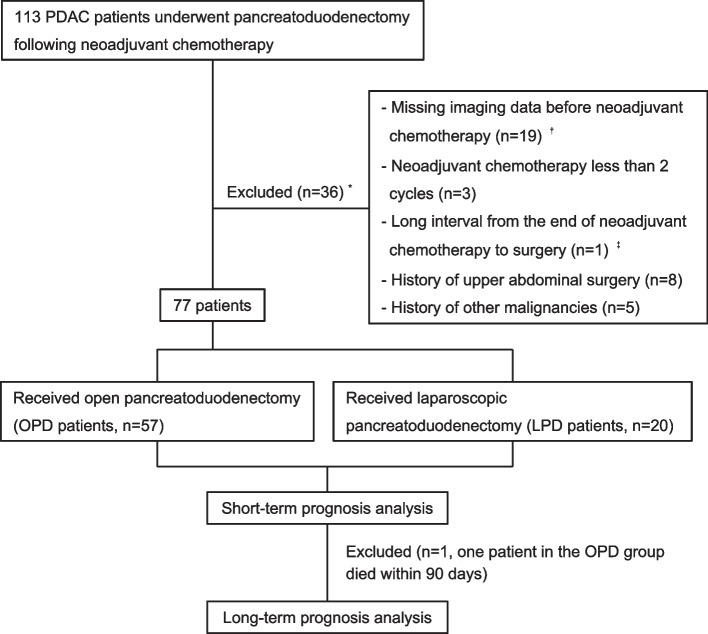
Flow chart of the study. Abbreviation: PDAC, pancreatic ductal adenocarcinoma; OPD, open pancreatoduodenectomy; LPD, laparoscopic pancreatoduodenectomy. *: only the main reason for exclusion is presented. †: due to the limitations of retrospective research, imaging data from other hospitals could not be obtained. ‡: the time interval between the end of neoadjuvant chemotherapy and the surgery in one patient was 74.6 weeks

The clinical data of 113 consecutive patients with borderline resectable PDAC who underwent pancreatoduodenectomy following NACT between January 2020 and December 2022 at Fudan University Shanghai Cancer Center was prospectively collected and retrospectively analyzed. Among them, those who had a history of other malignancies (*n* = 5), upper abdominal surgery (*n* = 8), incomplete imaging data (*n* = 19), < 2 cycles of NACT (*n* = 3), or an interval between the end of NACT and surgery > 12 weeks (*n* = 1) were excluded. The remaining 77 patients were included in the analysis, with 57 patients in the OPD group and 20 patients in the LPD group (Fig. 1). Except for NACT, none of the patients received any other antitumor treatment prior to the operation. The study was approved by the Shanghai Cancer Center Institutional Review Board and the requirement for individual consent was waived by the committee.

#### Neoadjuvant chemotherapy and efficacy evaluation

The resectability assessment of PDAC was conducted through multidisciplinary discussions in accordance with the National Comprehensive Cancer Network guideline (version 1. 2020). For anatomically borderline resectable and biologically borderline resectable (defined as resectable tumors with serum CA 19–9 levels ≥ 1000 U/mL) PDACs, NACT was routinely recommended and the regimens used in this study was nab-paclitaxel plus gemcitabine or modified FOLFIRINOX [12, 17–21]. Before initiating NACT, the pathological diagnosis of PDAC was confirmed through endoscopic ultrasonography-guided fine-needle biopsy or computed tomography-guided percutaneous fine-needle biopsy. For clinically diagnosed borderline resectable PDACs, NACT was administered after thoroughly informing them about the potential risks of inappropriate treatment and obtaining their informed consent. Postoperative histopathology confirmed the presence of PDAC in these patients.

In order to comprehensively assess the influence of NACT on surgical approaches, the duration of NACT in this study was set at a minimum of 2 cycles [16, 22]. Following every 2 treatment cycles, serum tumor markers and abdominal-enhanced computed tomography were reassessed. The efficacy of NACT was evaluated using changes in serum CA19-9 levels, the National Comprehensive Cancer Network guideline resectable status, and the response evaluation criteria in solid tumors (RECIST, version 1.1) [23].

#### Preoperative preparation and minimally invasive surgery

After NACT, in addition to thoracic and abdominal contrast-enhanced computed tomography and routine serological examination, contrast-enhanced magnetic resonance imaging or positron emission tomography scan was used to accurately assess tumor staging and exclude the presence of distant metastasis. For patients with biliary obstruction, percutaneous transhepatic cholangial drainage or endoscopic retrograde biliary drainage was employed during NACT treatment to alleviate jaundice and improve liver function. Metal biliary stents should be avoided, as they can exacerbate the local inflammatory response and affect the subsequent operation.

In this study, all LPD and OPD procedures were performed by four pancreatic surgeons in our center, each of whom had a minimum of 300 cases of OPD and 100 cases of LPD surgery experience, and had passed their respective learning curves. Surgical exploration for borderline resectable PDACs is scheduled within 4–8 weeks after completing NACT, as surgery beyond 8 weeks may be hindered by increased surgical difficulty due to local tumor fibrosis caused by NACT [13, 22]. Based on the adverse prognostic implication of elevated serum CA19-9 levels after NACT, surgical exploration is only recommended when the serum CA19-9 level remains stable or decreases [24, 25].

In addition to the LPD procedure being performed using a three-dimensional laparoscopic system and under constant pressure pneumoperitoneum conditions, all surgeons performed resection and reconstruction according to the same criteria. An anterior approach to the superior mesenteric artery (SMA) was employed to dissect the uncinate process, enabling early assessment of arterial involvement and preventing palliative resection [26–28]. Routine right-sided clearance of at least 180° of the SMA nerve plexus was performed [29]. If the superior mesenteric vein (SMV)/portal vein (PV) was involved, resection and reconstruction of the vein can be performed according to the International Study Group of Pancreatic Surgery classification of venous resections [30]. After specimen retrieval and meticulous hemostasis, gastrointestinal reconstruction was performed using the Child method. Specifically, an end-to-side duct-to-mucosal pancreaticojejunostomy was conducted, utilizing the modified Blumgart anastomosis technique as we previously reported [31]. A pancreatic duct stent was routinely placed and secured with sutures. Moreover, the pedicled teres ligament was employed to reinforce the posterior wall of the anastomosis and isolate the stump of the gastroduodenal artery [32].

#### Main outcome measures and follow-up

In the pathological examination conducted in this study, the resection margin referred to the pancreatic transection margin, bile duct margin, and stomach/duodenum margin. The margin status was assessed according to the criteria set by the Heidelberg Pancreatic Center. Specifically, an R0 resection was defined as the absence of tumor cells in the tissue ≤ 1 mm away from the margin under the microscope [33]. At our center, standard lymph node dissection is routinely performed during pancreaticoduodenectomy, which involves the removal of at least 15 lymph nodes.

Postoperative complications were defined as clinical events occurring within 90 days after the operation. Complications specific to pancreatic surgery, such as postoperative pancreatic fistula (POPF), postpancreatectomy hemorrhage, and delayed gastric emptying, were evaluated using the criteria of the International Study Group of Pancreatic Surgery [34–37]. The severity of complications was determined using the Clavien-Dindo classification system [38]. Hospital mortality was defined as death occurring within 90 days after the initial surgery [39]. All PDAC patients who underwent pancreatectomy after NACT were routinely given adjuvant chemotherapy after surgery and the regimen was adjusted based on previous NACT effectiveness and the patient's overall physical status.

Patient follow-up is conducted by a designated member of the surgical team. Enhanced computed tomography scans of the chest and abdomen, along with tumor marker examinations, are performed every 3 months within the first year after surgery as part of patient follow-up, and every 6 months after one year. If necessary, magnetic resonance imaging and/or positron emission tomography scans may be performed to clarify ambiguous computed tomography findings.

Recurrence-free survival (RFS) was defined as the duration from the time of surgery until the occurrence of tumor recurrence, patient death, or the last follow-up. Overall survival (OS) was defined as the length of time from the surgical procedure until the patient death or the last follow-up. Local recurrence was defined as recurrence within the surgical field, including tissue along the aorta, SMA, or celiac artery, as well as the soft tissue surrounding the biliary-jejunostomy or pancreaticojejunostomy. In this study, early recurrence was defined as recurrence within the first 6 months after surgery, as previous studies concluded that a recurrence-free interval of 6 months was the optimal threshold for distinguishing early from late recurrence after neoadjuvant therapy [40, 41].

#### Statistical analysis

Continuous variables were described using medians and quartiles and compared using Student's t-test or Mann–Whitney *U* test. Categorical variables were presented as frequencies and percentages and compared using the *χ*2 test or Fisher’s exact test. Logistic regression analyses were utilized to assess associations between underlying risk factors and early tumor recurrence, while Cox proportional hazards models were used to analyze RFS and OS outcomes. Survival rates were estimated using the Kaplan–Meier method and compared using the log-rank test. Two-sided *P* value < 0.05 were considered statistically significant. The statistical software utilized in this study included SPSS (23.0, SPSS Inc., Chicago, IL, United States) and R (4.2.1), with the R packages survival (3.3.1), survminer, and ggplot2 (3.3.6) loaded for analysis.

## Results

### Neoadjuvant chemotherapy and efficacy evaluation

The majority of patients (90.9%) had a preoperative pathological diagnosis of PDAC, with endoscopic ultrasonography-guided fine-needle biopsy (79.2%) being the most commonly used diagnostic method. Seven patients with clinically diagnosed PDAC refused biopsy and received NACT after signing informed consent. Eighty-seven percent of patients received NACT with nab-paclitaxel plus gemcitabine. The median number of treatment cycles in the OPD and LPD groups was 4 and 3, respectively (*P* = 0.008). The median time interval between the completion of NACT and the surgery was 4.4 (3.6–6.0) weeks in the whole cohort. Regarding NACT side effects, 80.5% of the patients experienced mild or no side effects, while 8 patients suffered from myelosuppression and 7 occurred gastrointestinal reactions (Table 1).

**Table 1:** Neoadjuvant chemotherapy and efficacy evaluation

**Variable**	**Number (%)/median (IQR)**			
	**Total (*n*=77)**	**OPD (n=57)**	**LPD (n=20)**	***P *value**
***Neoadjuvant chemotherapy information***				
**Pathological diagnosis method**				-
Endoscopic ultrasonography guided fine-needle biopsy	61 (79.2)	44 (77.2)	17 (85.0)	
Percutaneous fine needle biopsy	9 (11.7)	8 (14.0)	1 (5.0)	
Clinically diagnosed^a^	7 (9.1)	5 (8.8)	2 (10.0)	
**NACT regimens**				0.278
Nab-paclitaxel plus gemcitabine	67 (87.0)	51 (89.5)	16 (80.0)	
mFOLFIRINOX	10 (13.0)	6 (10.5)	4 (20.0)	
**NACT cycles**	3 (3-4)	4 (3-4)	3 (2-3)	**0.008**
**Time interval between the end of NACT and surgery**, weeks	4.4 (3.6-6.0)	4.3 (3.4-6.0)	4.7 (3.7-6.1)	0.654
**NACT side effects**				-
Myelosuppression	8 (10.4)	6 (10.5)	2 (10.0)	
Gastrointestinal reaction	7 (9.1)	6 (10.5)	1 (5.0)	
Mild or none side effects	62 (80.5)	45 (78.9)	17 (85.0)	
***Neoadjuvant chemotherapy efficacy evaluation***				
**CA19-9 before NACT**, U/mL	234.0 (60.0-607.0)	234.0 (56.0-590.0)	275.5 (86.7-672.5)	0.450
**CA19-9 after NACT**, U/mL	31.6 (16.0-128.0)	33.5 (16.0-170.0)	25.4 (16.5-98.0)	0.732
**Decrease rate of CA19-9**, %	70.4 (51.4-90.5)	66.9 (24.2-86.7)	85.3 (63.3-92.4)	**0.042**
**Tumor diameter before NACT**, cm	3.1 (2.4-3.6)	3.3 (2.4-3.7)	2.85 (2.4-3.6)	0.515
**Tumor diameter after NACT**, cm	2.5 (1.9-3.1)	2.6 (1.9-3.2)	2.2 (2.0-2.9)	0.277
**Shrinkage rate of diameter**, %	12.1 (6.7-31.0)	10.0 (5.9-30.0)	22.2 (11.5-32.3)	0.113
**SMA/CHA invasion before NACT**, yes	30 (39.0)	26 (45.6)	4 (20.0)	**0.043**
**SMV/PV invasion before NACT**, yes	60 (77.9)	45 (78.9)	15 (75.0)	0.758
**Resectable status before NACT**				0.107
Biologically borderline^b^	5 (6.5)	2 (3.5)	3 (15.0)	
Anatomically borderline	72 (93.5)	55 (96.5)	17 (85.0)	
**SMA/CHA invasion after NACT**, yes	25 (32.5)	23 (40.4)	2 (10.0)	**0.013**
**SMV/PV invasion after NACT**, yes	50 (64.9)	38 (66.7)	12 (60.0)	0.591
**Resectable status after NACT**				0.069
Anatomically resectable	13 (16.9)	7 (12.3)	6 (30.0)	
Anatomically borderline	64 (83.1)	50 (87.7)	14 (70.0)	
**RECIST status**				-
Partial response	23 (29.9)	15 (26.3)	8 (40.0)	
Stable disease	51 (66.2)	40 (70.2)	11 (55.0)	
Progressive disease^c^	3 (3.9)	2 (3.5)	1 (5.0)	

*Abbreviation*: *IQR* interquartile range, *OPD* open pancreatoduodenectomy, *LPD* laparoscopic pancreatoduodenectomy, *NACT* neoadjuvant chemotherapy, *SMA* superior mesenteric artery, *CHA* common hepatic artery, *SMV* superior mesenteric vein, *PV* portal vein, *RECIST* response evaluation criteria in solid tumors

^a^seven patients refused biopsy for pathological examination and were clinically diagnosed with pancreatic cancer by imaging and serological examination. Their postoperative pathological diagnosis was pancreatic ductal adenocarcinoma.

^b^anatomically resectable pancreatic cancer with serum CA19-9 level ≥ 1000 U/mL

^c^three patients were evaluated with progressive disease because the tumor diameter increased after NACT, they still received surgical exploration because the serum CA19-9 level decreased significantly

The median serum CA19-9 level before NACT was 234.0 (56.0–590.0) U/mL in the OPD group and 275.5 (86.7–672.5) U/mL in the LPD group (*P* = 0.450). After NACT, the median serum CA19-9 level decreased to 33.5 (16.0–170.0) U/mL in the OPD group and 25.4 (16.5–98.0) U/mL in the LPD group (*P* = 0.732), and the median decrease rate of CA19-9 in the LPD group was higher than that of the OPD group (85.3% vs. 66.9%, *P* = 0.042). The median tumor diameter before NACT was 3.3 (2.4–3.7) cm in the OPD group and 2.85 (2.4–3.6) cm in the LPD group (*P* = 0.515). Following NACT, the median tumor diameter decreased to 2.6 (1.9–3.2) cm in the OPD group and 2.2 (2.0–2.9) cm in the LPD group, with no significant difference observed in the diameter reduction rate (10.0% vs. 22.2%, *P* = 0.113).

**Fig. 2:**
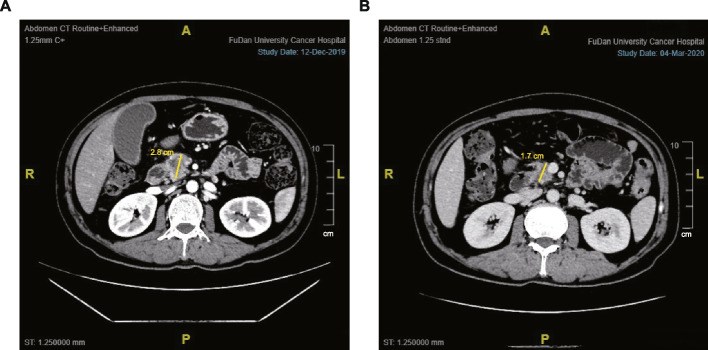
CT images of a patient who received 3 cycles of nab-paclitaxel plus gemcitabine regimen NACT, and the tumor was evaluated as partial response after the treatment. **A** CT image before NACT. **B** CT image after NACT. Abbreviation: NACT, neoadjuvant chemotherapy

The proportions of SMV/PV invasion did not show any significant differences between the OPD and LPD groups, both before and after neoadjuvant chemotherapy (NACT) (78.9% vs. 75.0%, *P* = 0.758; 66.7% vs. 60.0%, *P* = 0.591, respectively). However, the OPD group exhibited higher proportions of SMA/common hepatic artery (CHA) invasion compared to the LPD group, both before and after NACT (45.6% vs. 20.0%, *P* = 0.043; 40.4% vs. 10.0%, *P* = 0.013, respectively). There was no difference in the resectable status before NACT, with 55 (96.5%) anatomically borderline PDACs in the OPD group and 17 (85.0%) in the LPD group (*P* = 0.107). After NACT, 5 anatomically borderline PDACs in the OPD group and 3 in the LPD group were downstaged into resectable status (*P* = 0.069). Based on the RECIST criteria, 23 (29.9%) patients in the entire cohort were evaluated as having a partial response, while 51 (66.2%) were classified as having stable disease. Three patients were deemed to have progressive disease due to an increase in tumor diameter, but they still underwent surgical exploration because their serum CA19-9 levels had significantly decreased. Figure 2 depicted the case of one patient who received 3 cycles of nab-paclitaxel plus gemcitabine and exhibited a partial response after NACT.

### Preoperative and surgical information

Table 2 presents the preoperative and surgical information. The median age was 60.7 (54.1–67.2) years old, and the median body mass index was 21.9 (20.3–23.4) kg/m^2^. Among the patients, 20 (26.0%) patients had a history of diabetes mellitus. Additionally, 30 (39.0%) patients had a history of preoperative biliary drainage, with 24 of them undergoing percutaneous transhepatic cholangial drainage.

**Table 2:** Preoperative and surgical information

Variable	Number (%)/median (IQR)			
	Total (*n*=77)	OPD (*n*=57)	LPD (*n*=20)	*P *value
***Preoperative information***				
**Age**, years	60.7 (54.1-67.2)	59.7 (53.5-67.5)	61.4 (57.4-65.4)	0.763
**Gender**, male	36 (46.8)	27 (47.4)	9 (45.0)	0.855
**Body mass index**, kg/m^2^	21.9 (20.3-23.4)	21.3 (20.2-23.0)	22.0 (20.6-23.6)	0.387
**Diabetes mellitus**, yes	20 (26.0)	14 (24.6)	6 (30.0)	0.633
**Hypertension**, yes	17 (22.1)	12 (21.1)	5 (25.0)	0.714
**Biliary drainage**				-
Percutaneous transhepatic cholangial drainage	24 (31.2)	13 (22.8)	11 (55.0)	
Endoscopic retrograde biliary drainage	6 (7.8)	4 (7.0)	2 (10.0)	
No obstructive jaundice	47 (61.0)	40 (70.2)	7 (35.0)	
**White blood cell**, 10^^^9/L	6.0 (5.0-7.7)	6.0 (4.8-6.9)	7.2 (5.6-8.3)	**0.017**
**Hemoglobin**, g/L	117.0 (112.0-125.0)	117.0 (112.0-127.0)	116.5 (111.0-123.5)	0.659
**Alanine aminotransferase**, U/L	21.5 (14.1-39.4)	21.5 (13.6-43.7)	23.2 (16.5-33.8)	0.776
**Albumin**, g/L	43.4 (40.1-45.6)	43.5 (40.6-45.6)	42.0 (38.9-44.3)	0.106
**Total bilirubin**, μmol/L	8.4 (6.2-12.4)	8.1 (5.8-11.8)	10.3 (7.1-18.4)	0.158
**Prothrombin time**, seconds	12.2 (11.5-13.2)	12.2 (11.5-13.2)	12.5 (11.6-13.3)	0.630
**Serum creatinine**, μmol/L	58.0 (49.0-68.0)	61.0 (51.0-70.0)	51.5 (44.5-57.5)	**0.017**
***Surgical information***				
**ASA grade**				0.947
Grade I	8 (10.4)	6 (10.5)	2 (10.0)	
Grade II	69 (89.6)	51 (89.5)	18 (90.0)	
**Pancreatic duct diameter**, mm	3.6 (2.2-5.3)	3.4 (2.2-5.5)	3.8 (2.8-4.7)	0.650
**Operation time**, minutes	350 (302-415)	325 (295-378)	419 (375-468)	**<0.001**
**Blood loss**, mL	300 (200-600)	300 (200-600)	300 (200-500)	0.393
**Transfusion**, yes	28 (36.4)	21 (36.8)	7 (35.0)	0.883
**Superior mesenteric vein / Portal vein resection**, yes	32 (41.6)	25 (43.9)	7 (35.0)	0.489
**Type of venous resection**^**a**^				-
Partial venous excision with direct closure by suture closure	1 (1.3)	0 (0.0)	1 (5.0)	
Partial venous excision using a patch	2 (2.6)	1 (1.8)	1 (5.0)	
Segmental resection with primary venovenous anastomosis	18 (23.4)	14 (24.6)	4 (20.0)	
Segmental resection with interposed venous conduit and at least two anastomoses	11 (14.3)	10 (17.5)	1 (5.0)	

*Abbreviation*: *IQR* interquartile range, *OPD* open pancreatoduodenectomy, *LPD* laparoscopic pancreatoduodenectomy

^a^according to the International Study Group of Pancreatic Surgery classification of venous resections

The median operation time of the LPD group was longer than that of the OPD group (419 vs. 325 min, *P* < 0.001). There was no significant difference observed between the two groups in terms of pancreatic duct diameter (3.4 vs. 3.8 mm), blood loss amount (300 vs. 300 mL), transfusion rate (36.8% vs. 35.0%), and SMV/PV resection rate (43.9% vs. 35.0%). According to the International Study Group of Pancreatic Surgery classification of venous resections, segmental resection with primary venovenous anastomosis (23.4%) and segmental resection with interposed venous conduit and at least two anastomoses (14.3%) were the commonly used reconstruction methods.

### Postoperative pathological examination and complication

Twenty-nine (37.7%) PDACs were classified as having poor tumor differentiation. The two groups were comparable in the incidence of intravascular tumor thrombus (50.9% vs. 30.0%), perineural invasion (96.5% vs. 90.0%), adjacent tissue (78.9% vs. 70.0%) or organ (36.8% vs. 45.0%) invasion, and lymph node metastasis (54.4% vs. 45.0%). Specifically, there was no significant difference observed in the number of lymph nodes retrieved, with a median number of 22 in the OPD group and 18.5 in the LPD group (*P* = 0.393). In addition, the R1 margin rate (12.3% vs. 15.0%) and AJCC 8th TNM stage were also comparable between the two groups. (Table 3).

**Table 3:** Postoperative pathological examination and prognosis

Variable				Number (%)/median (IQR)			
				Total (*n*=77)	OPD (*n*=57)	LPD (*n*=20)	*P *value
***Postoperative pathological examination***							
**Tumor differentiation**							0.411
Poor				29 (37.7)	23 (40.4)	6 (30.0)	
Moderate or well				48 (62.3)	34 (59.6)	14 (70.0)	
**Intravascular tumor thrombus**, yes				35 (45.5)	29 (50.9)	6 (30.0)	0.107
**Perineural invasion**, yes				73 (94.8)	55 (96.5)	18 (90.0)	0.276
**Adjacent tissue invasion**, yes				59 (76.6)	45 (78.9)	14 (70.0)	0.416
**Adjacent organ invasion**, yes				30 (39.0)	21 (36.8)	9 (45.0)	0.520
**Lymph nodes retrieved**				22 (15-29)	22 (17-29)	18.5 (13-29)	0.393
**Lymph node metastasis**, yes				40 (51.9)	31 (54.4)	9 (45.0)	0.470
**R1 margin**, yes				10 (13.0)	7 (12.3)	3 (15.0)	0.756
**AJCC 8**^**th**^** T stage**							**0.013**
1-3				52 (67.5)	34 (59.6)	18 (90.0)	
4				25 (32.5)	23 (40.4)	2 (10.0)	
**AJCC 8**^**th**^** TNM stage**							0.141
I				25 (32.5)	15 (26.3)	10 (50.0)	
II				23 (29.9)	18 (31.6)	5 (25.0)	
III				29 (37.7)	24 (42.1)	5 (25.0)	
***Postoperative complication***							
**Postoperative length of stay**, days				12 (9-16)	12 (9-17)	12.5 (9-14.5)	0.714
**Clavien-Dindo classification**							**0.018**
None				32 (41.6)	19 (33.3)	13 (65.0)	
Yes		Grade 1		27 (35.1)	24 (42.1)	3 (15.0)	
		Grade 2		15 (19.5)	11 (19.3)	4 (20.0)	
		Grade ≥ 3		3 (3.9)	3 (5.3)	0 (0.0)	
**POPF**							0.174
None				47 (61.0)	32 (56.1)	15 (75.0)	
Yes		Biochemical		23 (29.9)	18 (31.6)	5 (25.0)	
		Grade B		6 (7.8)	6 (10.5)	0 (0.0)	
		Grade C		1 (1.3)	1 (1.8)	0 (0.0)	
**Hemorrhage**							0.177
None				71 (92.2)	54 (94.7)	17 (85.0)	
Yes	Degree		Mild	5 (6.5)	2 (3.5)	3 (15.0)	
			Sever	1 (1.3)	1 (1.8)	0 (0.0)	
	Time		Early	2 (2.6)	1 (1.8)	1 (5.0)	
			Late	4 (5.2)	2 (3.5)	2 (10.0)	
**Bile leakage**, yes				3 (3.9)	2 (3.5)	1 (5.0)	1.000
**Delayed gastric emptying**, yes				1 (1.3)	1 (1.8)	0 (0.0)	1.000
**Reoperation**, yes				1 (1.3)	1 (1.8)	0 (0.0)	1.000
**Death within 90 days**, yes				1 (1.3)	1 (1.8)	0 (0.0)	1.000
***Patterns of tumor recurrence (n=50)***							
**Time of recurrence**							0.764
Early recurrence				21 (42.0)	16 (43.2)	5 (38.5)	
Late recurrence				29 (58.0)	21 (56.8)	8 (61.5)	
**Site of recurrence**							0.356
Local				15 (30.0)	9 (24.3)	6 (46.2)	
Liver				15 (30.0)	12 (32.4)	3 (23.1)	
Multiple				20 (40.0)	16 (43.2)	4 (30.8)	

*Abbreviation*: *IQR* interquartile range, *OPD* open pancreatoduodenectomy, *LPD* laparoscopic pancreatoduodenectomy, *AJCC* American Joint Committee on Cancer, *POPF* postoperative pancreatic fistula

The median length of postoperative hospital stay was 12 days, with no significant difference observed between the groups (12.0 vs. 12.5 days, *P* = 0.714). However, the incidence of Clavien-Dindo complications (35.0% vs. 66.7%, *P* = 0.018) was significantly lower in the LPD group compared to the OPD group. None of the LPD patients experienced grade C POPF, severe hemorrhage, delayed gastric emptying, reoperation, or death within 90 days. In contrast, one patient in the OPD group suffered from grade C POPF combined with severe hemorrhage and died during the perioperative period after reoperation. Another patient in the OPD group experienced delayed gastric emptying.

### Postoperative prognosis

With a median follow-up time of 13.1 months, it was found that 50 patients (65.8%) experienced tumor recurrence. Out of these, 21 (42.0%) had early tumor recurrence, while the remaining 29 (58.0%) had late recurrence. In terms of the site of recurrence, 15 (30.0%) experienced local recurrences, 15 (30.0%) had liver metastases, and 20 (40.0%) had multiple recurrences (Table 3). The multivariable logistic regression analysis revealed that a decrease rate of CA19-9 > 70% (OR 0.309, 95% CI 0.099–0.960,* P* = 0.042) was identified as a protective factor against early tumor recurrence, whereas poor tumor differentiation was associated with an increased risk (OR 3.805, 95% CI 1.271–11.393, *P* = 0.017) (Table 4).

**Table 4:** Regression analysis of early tumor recurrence and recurrence free survival (*n*=76)

**Variable**	**Logistic regression analysis of early tumor recurrence**						**Cox regression analysis of recurrence free survival**					
	**OR**	**95% CI**	***P*** **value**	**OR**	**95% CI**	***P*** **value**	**HR**	**95% CI**	***P*** **value**	**HR**	**95% CI**	***P*** **value**
**Age**, years (continuous)	0.997	0.943-1.054	0.916				0.985	0.957-1.014	0.306			
**Gender**, male vs. female	1.320	0.482-3.615	0.589				0.889	0.503-1.570	0.684			
**Diabetes mellitus**, yes vs. no	0.373	0.097-1.436	0.152				0.710	0.353-1.427	0.336			
**Decrease rate of CA19-9**, %, > vs. ≤70	0.267	0.090-0.793	**0.017**	0.309	0.099-0.960	**0.042**	0.578	0.325-1.030	0.063			
**Tumor diameter before NACT**, cm, > vs. ≤3	1.812	0.649-5.065	0.257				2.189	1.174-4.082	**0.014**	2.185	1.166-4.097	**0.015**
**RECIST status**, partial response vs. stable disease or progressive disease	1.641	0.567-4.746	0.361				1.171	0.651-2.108	0.598			
**Surgical approach**, LPD vs. OPD	0.833	0.260-2.675	0.759				0.715	0.375-1.360	0.306			
**Transfusion**, yes vs. no	1.166	0.412-3.304	0.773				1.321	0.728-2.396	0.360			
**Superior mesenteric vein / Portal vein resection**, yes vs. no	2.333	0.838-6.497	0.105				1.681	0.952-2.969	0.074			
**Tumor differentiation**, poor vs. moderate or well	4.333	1.498-12.532	**0.007**	3.805	1.271-11.393	**0.017**	1.819	1.039-3.184	**0.036**	1.805	1.030-3.163	**0.039**
**Intravascular tumor thrombus**, yes vs. no	1.174	0.428-3.220	0.755				1.535	0.865-2.726	0.143			
**Adjacent tissue invasion**, yes vs. no	1.451	0.417-5.049	0.558				1.521	0.775-2.987	0.223			
**Adjacent organ invasion**, yes vs. no	1.312	0.471-3.655	0.603				0.968	0.546-1.715	0.912			
**Lymph node metastasis**, yes vs. no	1.061	0.388-2.902	0.909				1.500	0.854-2.637	0.159			
**R1 margin**, yes vs. no	1.922	0.483-7.640	0.354				1.609	0.713-3.630	0.252			

*Abbreviation*:* OR* odds ratio, *CI* confidence interval, *HR* hazard ratio, *NACT* neoadjuvant chemotherapy, *RECIST* response evaluation criteria in solid tumors, *LPD* laparoscopic pancreatoduodenectomy, *OPD* open pancreatoduodenectomy

**Fig. 3:**
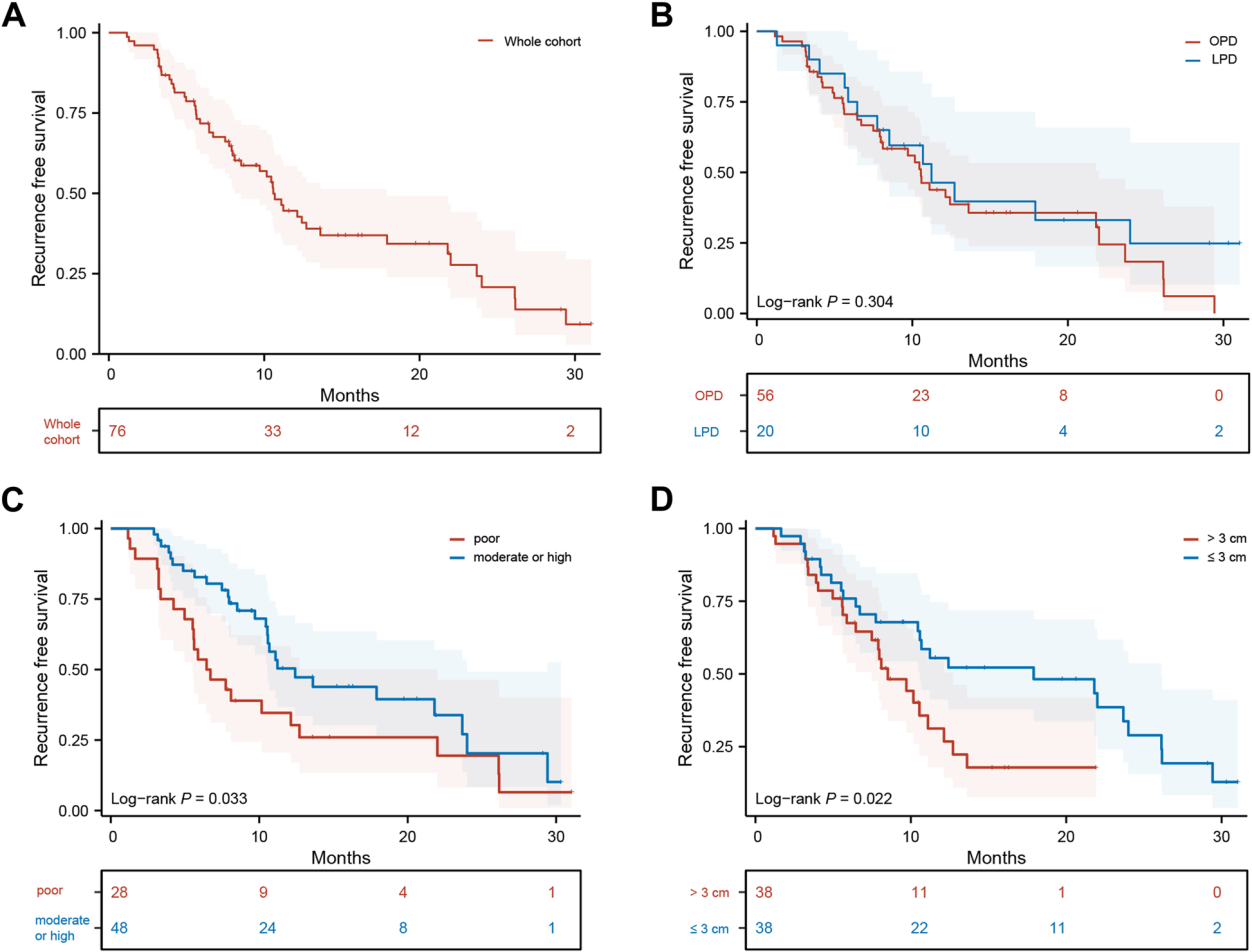
Recurrence-free survival of borderline resectable PDAC following NACT after pancreatoduodenectomy. **A** The whole cohort. **B** OPD versus LPD. **C** poor tumor differentiation versus moderate or high. **D** Tumor diameter > 3 cm before NACT versus ≤ 3 cm. Abbreviation: PDAC, pancreatic ductal adenocarcinoma; NACT, neoadjuvant chemotherapy; OPD, open pancreatoduodenectomy; LPD, laparoscopic pancreatoduodenectomy

The median RFS for the whole cohort was 10.6 months, with a median RFS of 11.2 months for the LPD group and 10.5 months for the OPD group, showing no significant difference between the two groups (*P* = 0.304, Fig. 3A, B). Notably, patients with poor tumor differentiation (6.6 vs. 12.4 months, *P* = 0.033), or a tumor diameter > 3 cm before NACT (8.5 vs. 17.9 months, *P* = 0.022), had a worse RFS (Fig. 3C, D). The multivariable Cox regression analysis showed that a tumor diameter > 3 cm before NACT (HR 2.185, 95% CI 1.166–4.097, *P* = 0.015) and poor tumor differentiation (HR 1.805, 95% CI 1.030–3.163, *P* = 0.039) were independent risk factors for RFS, while the surgical approach did not have a significant impact on postoperative RFS in patients following NACT (Table 4). During the limited follow-up period of this study, no differences were observed in OS between the LPD and OPD groups (*P* = 0.304, Supplementary Figure S1A). The multivariable Cox regression analysis revealed that a decrease rate of CA19-9 > 70% was associated with a protective effect on OS (HR 0.322, 95% CI 0.121–0.855, *P* = 0.023) (Supplementary Table S1, Supplementary Figure S1B).

## Discussion

In this study, we found no significant difference in RFS and OS between patients who underwent LPD and those who underwent OPD for borderline resectable PDAC after NACT. Although LPD was associated with prolonged operation time, the incidence of Clavien-Dindo complications was lower. Effective control of CA19-9 levels (decrease rate > 70%) can help reduce the risk of early postoperative tumor recurrence and improve OS. To the best of our knowledge, this is the first report investigating the oncological outcomes and prognosis of patients with PDAC following NACT who subsequently underwent LPD compared to OPD.

Studies have established the safety and oncological equivalence of minimally invasive pancreatomy for PDACs, with some suggesting a better prognosis compared to open surgery [2–8]. However, due to the limited number of patients eligible for upfront resection [9], and the value of NACT in improving the prognosis of borderline resectable PDAC [10–16], further exploration is urgently needed to determine the feasibility of using minimally invasive techniques in these patients. PDAC patients requiring NACT often present with a high tumor burden and close association between the tumor and mesenteric vasculature, frequently accompanied by tissue inflammation and edema. Furthermore, due to the rich stromal content of PDACs, radiologic anatomical downstaging is uncommon [42]. In a RECIST evaluation of 129 borderline resectable PDACs at MD Anderson Cancer Center, only 15 cases (12%) were deemed to have a partial response, and there was no significant association between RECIST response and postoperative survival [43]. Similarly, in this study, only 23 patients (29.9%) achieved a partial response, and 8 patients (11.1%) had their resectable status downstaged from anatomically borderline to anatomically resectable. Furthermore, there was no statistically significant difference between the LPD group and the OPD group. Therefore, LPD surgery in these patients is a highly challenging and risky procedure, requiring significant demands on the surgical team to safely remove the tumor and ensure optimal oncological outcomes.

Inflammation and fibrosis of the pancreatic tissue caused by chemotherapy reactions and obstructive pancreatitis from the tumor can lead to bleeding during LPD surgery, potentially affecting the surgeon’s view and requiring conversion to open surgery. To address this, proper coordination of the suction device and the utilization of bipolar electrocoagulation are recommended to maintain a clear surgical field. During anatomical dissection, it is advisable to follow the “Easy First” principle by selecting an area with minimal inflammation and optimal tissue structure for initial entry into the vascular layer [44]. Excising the uncinate process following NACT presents a challenge in LPD. The tumor often densely adheres to or invades the mesenteric vein in these cases, and improper separation can result in uncontrolled major bleeding. To minimize complications, the artery-first approach is recommended during this procedure [26]. In the resection process, splenic vein disconnection may be considered if necessary. However, it should not be routine due to the potential for clinically significant left-sided portal hypertension in 29.4% of cases [45, 46]. When PDAC involves SMV/PV, laparoscopic vascular resection and reconstruction can be performed. Ensuring a smooth venous intima and minimizing vascular tension is crucial in preventing thrombosis after reconstruction [47]. In the current study, all 20 patients in the LPD group successfully completed the operation without conversion to open surgery, although the operation time was relatively prolonged. Twenty-five (43.9%) and seven (35.0%) patients in the LPD group and OPD group received SMV/PV resection, respectively (*P* = 0.489). Segmental resection with primary venovenous anastomosis was the most common type of revascularization, accounting for 23.4% of the entire cohort (Supplementary Video S1).

Neoadjuvant therapy can inhibit tumor growth, resulting in a better R0 resection rate and a lower lymph node-positive rate [10–13]. Although the rates of radiological tumor downstaging may be low, the incidence of negative surgical margins is high. A previous study has demonstrated that 94% of patients are able to achieve R0 margins [42]. In this study, both the OPD and LPD groups achieved favorable R0 margin rates of 87.7% and 85.0%, respectively (*P* = 0.756). In addition to surgical margin status, the number of retrieved lymph nodes serves as a surrogate indicator of surgical oncologic adequacy. The median number of harvested lymph nodes in the LPD group was 18.5, exceeding the standard requirement of 15 for pancreatoduodenectomy and not statistically different from the OPD group. These preliminary data suggest that LPD for PDAC after NACT is oncologically equivalent to OPD.

A prospective multicenter study analyzed the impact of neoadjuvant radiochemotherapy on the prognosis of borderline resectable PDAC and found that neoadjuvant treatment followed by surgery was not associated with a higher incidence of POPF, delayed gastric emptying, wound infection, or other complications compared to upfront surgery [13]. Although our study did not find a significant difference between LPD and OPD in terms of complications specific to pancreatic surgery, including POPF, hemorrhage, bile leakage, and delayed gastric emptying, the LPD group exhibited a lower incidence of Clavien-Dindo complications compared to the OPD group (35.0% vs. 66.7%, *P* = 0.018). With the implementation of modern surgical techniques focused on enhanced recovery after surgery, the benefits of minimally invasive procedures in terms of postoperative hospitalization duration have gradually diminished. In this study, the median postoperative length of stay for patients in both groups was 12 days. It should be noted that one patient in the OPD group experienced grade C POPF accompanied by severe hemorrhage and died during the perioperative period after reoperation, and one patient suffered from delayed gastric emptying.

After a median follow-up of 13.1 months, we observed that the LPD group achieved comparable RFS and OS rates to the OPD group. Tumor recurrence was observed in 50 out of 76 patients (65.8%), with 21 (42.0%) of them experiencing early tumor recurrence. Predicting early tumor recurrence after surgery plays a crucial role in determining the appropriate surgical strategy for borderline resectable PDAC following NACT. Previous studies have analyzed factors influencing early recurrence after surgery for resectable and locally advanced PDAC following induction therapy [40, 41, 48]. However, there have been no reports specifically focusing on borderline resectable PDAC following NACT. Our study revealed that a decrease rate of CA19-9 > 70% after NACT (OR 0.309) was identified as a protective factor against early tumor recurrence. This finding is consistent with previous studies that have demonstrated the prognostic value of CA19-9, where the normalization of serum CA19-9 following neoadjuvant therapy is considered the strongest predictor of long-term survival [24]. Furthermore, our multivariable regression analysis indicated the protective effect of controlling CA19-9 levels on OS (HR 0.322). Therefore, we conclude that in the current era of effective NACT, relying solely on anatomic criteria is insufficient to define resectability for PDAC. Instead, a novel prognostic-based classification system that incorporates tumor biology and response to NACT should be developed to establish a more accurate foundation for defining resectability [49].

The results of this study demonstrate the feasibility of LPD for borderline resectable PDAC following NACT. However, technical capability does not equate to necessity in performing a risky surgery. The primary focus for borderline resectable PDACs after NACT should be on achieving radical resection while prioritizing the well-being and best interests of the patient. Due to the inherent limitations of retrospective studies and the small sample size, it is necessary to conduct larger cohort studies or prospective randomized controlled clinical trials to validate whether LPD can provide oncological benefits for these patients. Furthermore, it is worth noting that in this study, the OPD group had a higher proportion of tumors classified as T4 stage due to involvement of the SMA/CHA (40.4% vs. 10.0%, *P* = 0.013). The results suggest that the presence of arterial involvement by the tumor is a key factor influencing the choice between OPD or LPD for surgeons, although arterial resection and reconstruction were not performed in this study. Considering the potential surgical risks involved in LPD and the requirement for surgeons to have significant experience, it is advisable to conduct such studies exclusively in large pancreatic surgery centers.

## Conclusions

LPD is a feasible and oncologically equivalent option for treating PDAC following NACT compared to OPD. Despite the prolonged operation time, LPD has advantages in reducing overall postoperative complications. Effective control of serum CA19-9 levels is beneficial in reducing the risk of early tumor recurrence and improving OS.

### Supplementary Information

**Additional file 1: Figure S1.**

**Additional file 2: Table S1.**

**Additional file 3.** 

## Data Availability

The datasets used and/or analyzed during the current study are available from the corresponding author upon reasonable request (Xiaowu Xu. Emails: xuxiaowu@fudanpci.org).
