# Supplementary figures and images for: Feasibility of laparoscopic versus open pancreatoduodenectomy following neoadjuvant chemotherapy for borderline resectable pancreatic cancer: a retrospective cohort study
Source: World J Surg Oncol. 2024 Jan 2;22:1. doi: 10.1186/s12957-023-03277-2 (PMC10759588; doi:10.1186/s12957-023-03277-2)

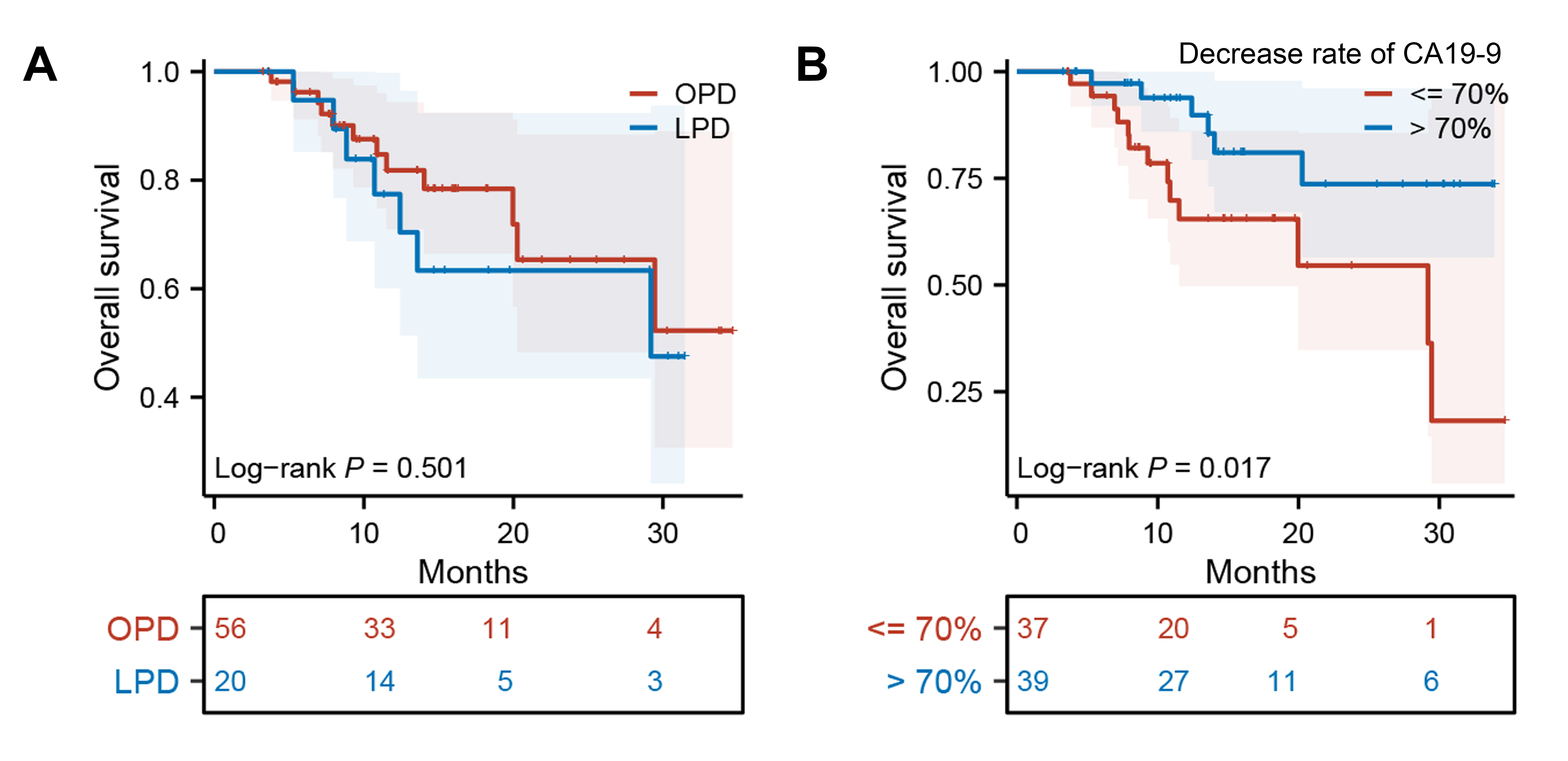

Supplement: Supplementary file 1 — Additional file 1: Figure S1. [file 12957_2023_3277_MOESM1_ESM.jpg]
