# Supplementary material for: Feasibility of laparoscopic versus open pancreatoduodenectomy following neoadjuvant chemotherapy for borderline resectable pancreatic cancer: a retrospective cohort study
Source: World J Surg Oncol. 2024 Jan 2;22:1. doi: 10.1186/s12957-023-03277-2 (PMC10759588; doi:10.1186/s12957-023-03277-2)
Supplement: Supplementary file 2 — Additional file 2: Table S1. [file 12957_2023_3277_MOESM2_ESM.docx]

| **Supplementary Table 1. Cox regression analysis of** **overall survival (n=76)** | | | | | | |
| --- | --- | --- | --- | --- | --- | --- |
| **Variable** | **Uni****variate** | | | **Multivariate** | | |
|  | **HR** | **95% CI** | ***P***  **value** | **HR** | **95% CI** | ***P***  **value** |
| **Age**, years (continuous) | 1.009 | 0.960-1.059 | 0.729 |  |  |  |
| **Gender**, male vs. female | 1.618 | 0.653-4.008 | 0.299 |  |  |  |
| **Diabetes mellitus**, yes vs. no | 0.578 | 0.167-1.995 | 0.386 |  |  |  |
| **Decrease rate of CA19-9**, %, > vs. ≤70 | 0.322 | 0.121-0.855 | **0.023** | 0.322 | 0.121-0.855 | **0.023** |
| **Tumor diameter before NACT**, cm, > vs. ≤3 | 2.059 | 0.766-5.533 | 0.152 |  |  |  |
| **RECIST status,** partial response vs. stable disease or progressive disease | 0.800 | 0.303-2.116 | 0.654 |  |  |  |
| **Surgical approach**, LPD vs. OPD | 1.377 | 0.540-3.512 | 0.503 |  |  |  |
| **Transfusion**, yes vs. no | 1.404 | 0.524-3.762 | 0.500 |  |  |  |
| **Superior mesenteric vein / Portal vein resection**, yes vs. no | 1.643 | 0.641-4.212 | 0.302 |  |  |  |
| **Tumor differentiation**, poor vs. moderate or well | 1.587 | 0.643-3.922 | 0.317 |  |  |  |
| **Intravascular tumor thrombus**, yes vs. no | 1.493 | 0.587-3.796 | 0.400 |  |  |  |
| **Adjacent tissue invasion**, yes vs. no | 1.201 | 0.431-3.348 | 0.727 |  |  |  |
| **Adjacent organ invasion**, yes vs. no | 1.122 | 0.440-2.862 | 0.810 |  |  |  |
| **Lymph node metastasis**, yes vs. no | 1.296 | 0.519-3.236 | 0.579 |  |  |  |
| **R1 margin**, yes vs. no | 0.934 | 0.213-4.103 | 0.928 |  |  |  |

Abbreviation: HR, hazard ratio; CI, confidence interval; NACT, neoadjuvant chemotherapy; RECIST, response evaluation criteria in solid tumors; LPD, laparoscopic pancreatoduodenectomy; OPD, open pancreatoduodenectomy.
